# The solid-state conformation of the topical anti­fungal agent *O*-naphthalen-2-yl *N*-methyl-*N*-(3-methyl­phen­yl)carbamo­thio­ate

**DOI:** 10.1107/S2053229618013591

**Published:** 2018-10-23

**Authors:** Douglas M. Ho, Michael J. Zdilla

**Affiliations:** a Department of Chemistry, Temple University, 1901 N. 13th St., Philadelphia, PA 19122, USA

**Keywords:** tolnaftate, topical anti­fungal, solid-state conformation, crystal structure

## Abstract

Tolnaftate, a classic anti­fungal compound, has been found to crystallize from 1:1 (*v*/*v*) acetone–water as large flat colorless needles in the centrosymmetric monoclinic space group *P*2_1_/*c*. These crystals contain a 50:50 mixture of the (+*ap*,−*sp*,+*ac*,−*ac*) and (−*ap*,+*sp*,−*ac*,+*ac*) conformers. The bond lengths in the central CNOS unit are 1.3444 (19), 1.3556 (18) and 1.6567 (15) Å for C—N, C—O and C—S, respectively, and the CNOS and C_3_N moieties are flat and nearly coplanar with each other, consistent with the C—N bond possessing partial double-bond character. Tolnaftate and the four most closely related *N*,*N*-di­substituted thio­carbamates in the Cambridge Structural Database (CSD) all exist as *E*-conformational isomers in the solid state. Among these five compounds, tolnaftate is the only one in which the *N*-tolyl moiety is positioned *trans* to the S atom, *i.e.* the *N*-aryl substituent in each of the other compounds is positioned *cis* to their respective S atom. Notably, and more importantly, our *experimental* X-ray structure is unlike all prior *theoretical* models available for tolnaftate. The implication, either directly or indirectly, is that some of those theoretical models used in earlier studies to explain the spectroscopic properties of tolnaftate and to suggest which protein–ligand inter­actions are responsible for the binding of tolnaftate to squalene epoxidase are either inappropriate or structurally unreasonable, *i.e.* the results and conclusions from those prior studies are in need of critical reassessment.

## Abstract 2

Large flat colorless needles of tolnaftate have been obtained from Tinactin, and have been shown to contain a 50:50 mixture of the (+*ap*,−*sp*,+*ac*,−*ac*) and (−*ap*,+*sp*,−*ac*,+*ac*) conformers. This finding fills a long-standing void in the structural chemistry of this classic anti­fungal compound, and suggests that prior theoretical models used to explain the spectroscopy, bioactivity, and mode of binding of tolnaftate to squalene epoxidase are probably inappropriate or suspect.

## Introduction   

*O*-Naphthalen-2-yl *N*-methyl-*N*-(3-methyl­phen­yl)carbamo­thi­o­ate, (I), is a synthetic thio­carbamate from the 1960s with anti­mycotic activity (Noguchi *et al.*, 1961 ▸, 1963 ▸). It is perhaps most readily recognized by the generic name tolnaftate and is primarily used to treat fungal skin infections, such as athlete’s foot (tinea pedis), jock itch (tinea cruris), and ringworm (tinea capitis and tinea corporis). Tolnaftate is a squalene epoxidase inhibitor used to disrupt the biosynthesis of ergosterol, resulting in a toxic accumulation of squalene and ultimately fungal cell death (Morita & Nozawa, 1985 ▸; Ryder *et al.*, 1986 ▸; Barrett-Bee *et al.*, 1986 ▸). It was launched for human use in 1965 by Schering Corporation as the active pharmaceutical ingredient (API) in Tinactin (Sittig, 1988 ▸). Schering Corporation subsequently merged with Plough in 1971 and Merck in 2009. Today, Tinactin is marketed by Bayer which acquired Merck’s consumer care products in 2014. Tolnaftate is present in numerous anti­fungal products worldwide either as the sole active ingredient or in combination with one or more other APIs.

Historically, tolnaftate was introduced after griseofulvin, a natural product isolated from the mycelium of *Penicillium griseofulvum* (Oxford *et al.*, 1939 ▸). Griseofulvin was launched in 1959 by McNeil, Schering and Ayerst (Sittig, 1988 ▸) and is widely held to have been the first globally successful commercial anti­fungal agent. Griseofulvin is administered orally (being topically ineffective) and adverse side effects, *e.g.* photosensitivity, nausea, headaches, insomnia and so on, while infrequent, have been noted. In contrast, tolnaftate is administered topically (being orally ineffective) with little to no side effects and holds the distinction of being the first globally successful *synthetic topical* anti­fungal agent (Robinson & Raskin, 1964 ▸). Other popular topical anti­fungal compounds would be launched years later, *e.g.* clotrimazole, miconazole nitrate, terbinafine hydro­chloride, and butenafine hydro­chloride in 1973, 1974, 1991, and 1992, respectively (Sittig, 1988 ▸; Newman & Cragg, 2016 ▸). That tolnaftate has maintained a presence in the global over-the-counter marketplace in spite of the development of these newer APIs is rather remarkable.
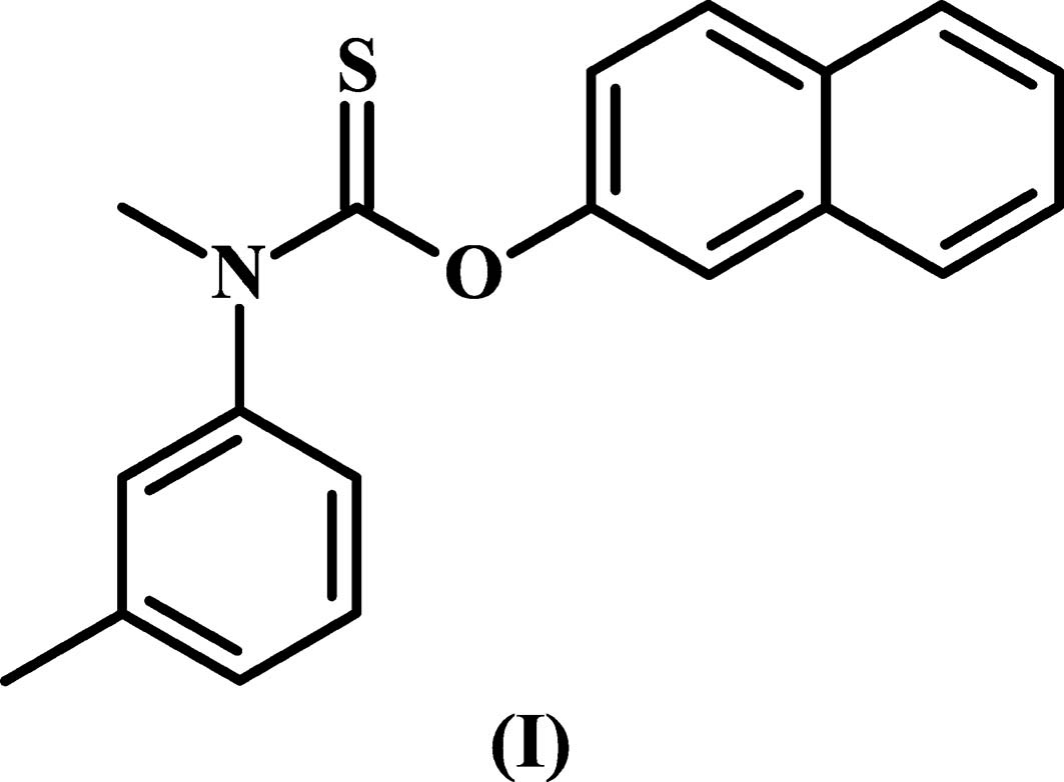


Crystallographically, griseofulvin, clotrimazole, miconazole nitrate, and the hydro­chloride salts of terbinafine and butenafine have all been structurally characterized. For example, the Cambridge Structural Database (CSD; Groom *et al.*, 2016 ▸) lists 13 entries for griseofulvin alone, with the two most recent studies having been published earlier this year (Mahieu *et al.*, 2018 ▸; Su *et al.*, 2018 ▸). However, the crystal structure of tolnaftate, an equally classic compound as griseofulvin in the anti­fungal arena, is nowhere to be found in the CSD or the scientific literature. We therefore felt compelled to remedy this long-standing oversight of this historic compound that has provided relief to so many of us over the past half century.

## Experimental   

### Isolation and crystallization   

A small vial was charged with 2 ml of Bayer Tinactin Liquid Spray followed by 2 ml of water and sealed. Upon standing at room temperature, extremely tiny colorless needles of tolnaftate formed and were harvested. These were redissolved in a minimum amount of 1:1 (*v*/*v*) acetone–water and the capped vial of the resultant solution then placed in a freezer to effect supersaturation and nucleation. As soon as crystals were noted, the vial was removed from the freezer and allowed to warm to room temperature, at which point the vial cap was loosened and the acetone–water allowed to evaporate slowly further to yield large flat colorless needles suitable for a single-crystal X-ray diffraction experiment. No attempt was made to optimize the acetone–water ratio or to try other solvents since the task of obtaining crystallographic quality crystals had been achieved.

### Refinement   

Crystal data, data collection and structure refinement details are summarized in Table 1 ▸. A riding model was used for the H atoms, with the C—H distances constrained to 0.95 and 0.98 Å for the aryl and methyl moieties, respectively, and the *U*_iso_(H) values set at 1.2*U*_eq_(C) and 1.5*U*_eq_(C) for the aryl and methyl H atoms, respectively. The *m*-tolyl methyl group was treated as rotationally disordered over two orientations. The refined site-occupancy factors were 0.76 (2) and 0.24 (2) for the major and minor components of that disorder, respectively.

## Results and discussion   

Some readers may have already surmised from §1 and §2.1, that this study is a spin-off from a STEM outreach project for informal chemical and crystallographic education, *i.e.* for grades 6–12 pre-college students, homeschoolers, hobbyists, and amateur scientists. Chemistry is often introduced to this audience in the digestible and relatable form of common mol­ecules and common household chemicals. One of the design criteria for our outreach project was to base it on a *less commonly* recognized common mol­ecule. We believe that tolnaftate fits that criterion. It has been found in numerous households for over 50 years, yet most individuals have no notion of its structural identity and make-up. Another design criterion was cost, *i.e.* the chemical source was required to be relatively inexpensive and readily available to the targeted audience. The Bayer Tinactin Liquid Spray used in this study was purchased from a retail store for less than 6 US dollars. Other generic sprays can also be purchased for 3–4 US dollars. A single can of Tinactin can provide 2 ml aliquots for a class of roughly 75 students at a cost of 8 cents per student (or 150 students at 4 cents per student, if they work in pairs). We hope that the results and discussion provided below will be educational and simulating for those inter­ested in STEM, and meaningful and entertaining for our academic and industrial colleagues as well.

### Experimentally observed conformation   

**Figure 1:**
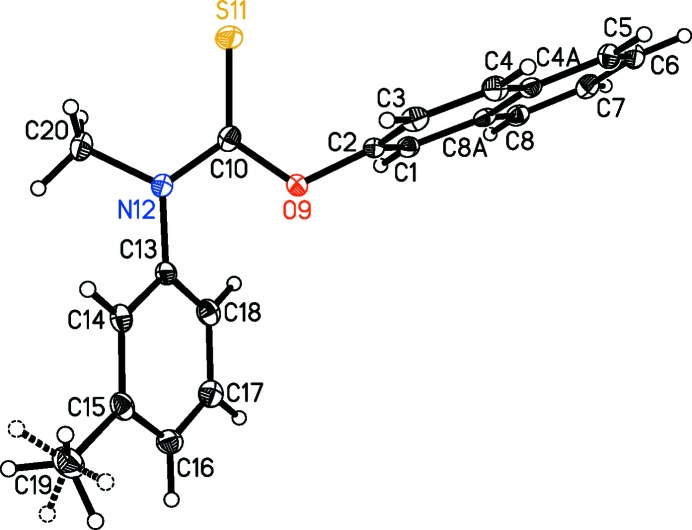
The mol­ecular structure of (I), showing the atom-labeling scheme. Displacement ellipsoids are drawn at the 50% probability level. The minor component of the disordered tolyl methyl group is drawn with dashed circles for the H atoms and dashed C—H bonds.

The mol­ecular structure of (I) is shown in Fig. 1 ▸. As a thio­carbamate, the geometric parameters of inter­est to most readers are those associated with the CNOS core. The O9—C10, C10—S11, and C10—N12 bond lengths are 1.3556 (18), 1.6567 (15), and 1.3444 (19) Å, respectively, and are in excellent agreement with the literature values of 1.360 (11), 1.671 (24), and 1.346 (23) Å for C*sp* ^2^—O, C*sp* ^2^=S, and C*sp* ^2^—N*sp* ^2^ bonds, respectively (Allen *et al.*, 1987 ▸). For comparison, the literature values for C*sp* ^2^—S and C*sp* ^2^—N*sp* ^3^ bonds are 1.751 (17) and 1.416 (18) Å, respectively. The sums of the bond angles at atoms C10 and N12 are 359.98 (13) and 359.97 (13)°, respectively, and are also consistent with those atoms being formally *sp* ^2^-hydridized. Individually, however, the bond angles at C10 do exhibit significant deviations from the idealized *sp* ^2^ value of 120°, *e.g.* the O9—C10—S11, O9—C10—N12, and S11—C10—N12 angles are 124.48 (11), 110.39 (13), and 125.11 (12)°, respectively. This pattern of two angles exceeding 120° and the third angle encroaching on the idealized *sp* ^3^ value of 109.5° is commonly observed in thio­carbamates (and even carbamates).

The three substituents attached to the CNOS core exhibit the expected structural metrics. The aromatic rings are flat, with the r.m.s. deviations for the planes defined by atoms C1–C4/C4*A*/C5–C8/C8*A* and C13–C18 both being 0.0106 Å. The C2—O9—C10 angle is 119.25 (11) *versus* 120.0° for an idealized O*sp* ^2^ atom, the C10/N12/C13/C20 moiety and the CNOS core are both planar, with r.m.s. deviations of 0.0060 and 0.0053 Å for the fitted atoms defining each plane, and the C2, C13, and C20 atoms are 0.091 (2), 0.009 (2), and −0.074 (3) Å off of the CNOS plane, respectively. The CNOS and C_3_N moieties are also nearly coplanar with each other, with the angle between their normals being 2.24 (11)°. These observations suggest that delocalization of π-electron density over the entire CNOS unit is not geometrically disallowed or, at least, that the core C—N bond possesses partial double-bond character.

As depicted in the Scheme and Fig. 1 ▸, to a first approximation, the tolnaftate mol­ecule is present in an *E* conformation in the solid state. The four most closely related *N*,*N*-disubstituted thio­carbamate structures in the CSD are GEHSAO (Mugnoli *et al.*, 2006 ▸), JOXQIW (Sakamoto *et al.*, 1998 ▸), MESHAY (Bowman *et al.*, 2007 ▸), and YEDRAA (Vovk *et al.*, 1992 ▸). In these, the methyl group is replaced by a C(=*X*)*R* group, with *X* being either an O or S atom, and the aryl substitutent is either a tolyl or a phenyl group. The mol­ecules in these prior structures are also present as *E* conformers. A wider comparison involving all relevant mono­substituted *N*-aryl thio­carbamates, *i.e.* with the methyl group replaced by an H atom, yields 46 such entries in the CSD with the ratio of *E*:*Z* stereochemistries being 40:6. Obviously, the *N*,*N*-disubstituted comparison suffers from both steric and electronic factors, *e.g.* the C(=*X*)*R* groups are significantly larger and more polar than methyl, and the monosubstituted comparison suffers from N—H being significantly more prone to hydrogen-bonding effects than N—CH_3_. Nevertheless, prior studies would seem to suggest that an *E* conformation is preferred, and that is indeed what is found for tolnaftate as well.

For casual readers, this first approximation for describing the tolnaftate mol­ecule is more than adequate. For others, additional stereodescriptors are required. Readers in the latter group will point out that Fig. 1 ▸ also clearly shows that the naphthyl moiety is *cis* to the S atom, *i.e. s–cis* with respect to the C—O core bond, and that a more precise description of the tolnaftate mol­ecule is that it has an *E*,*Z* or *trans*,*cis* conformation. While justifiably superior to the *E*-only description, this second approximation using two stereodescriptors rather than one also falls short of being fully descriptive. For example, the tolnaftate mol­ecule shown is three-dimensional (3D) and chiral in the solid state, *i.e.* the conformer in Fig. 1 ▸ and its enanti­omer are present in our crystal as a 50:50 racemic mixture, and therein lies the problem. While the inverted mol­ecule is indeed nonsuperposable on the conformer in Fig. 1 ▸, that enanti­omer would be assigned the exact same stereodescriptors, *i.e.* it too is an *E*,*Z* or *trans*,*cis* conformer. Hence, one cannot differentiate between the two enanti­omers with these stereodescriptors because the descriptors themselves are invariant on reflection in a mirror.

A third approach for specifying the conformation is to use clinal and periplanar descriptors, *i.e.* https://doi.org/10.1351/goldbook.T06406 (Klyne & Prelog, 1960 ▸; Moss, 1996 ▸). These offer two significant advantages over the *Z*/*E* and *cis*/*trans* nomenclature, *i.e.* (*a*) they divide torsional space into six 60° regions rather than two 180° semicircular sections, and (*b*) they are signed + or −. We will apply a few nonstandard conventions and further subdivide the +30 to −30° region into 0 to +30° and 0 to −30° and assign the descriptors +*sp* and −*sp* to them. Similarly, the +150 to −150° zone will be subdivided into 180 to +150° and 180 to −150° and assigned the descriptors +*ap* and −*ap*, respectively. This subdivision of torsional space into eight regions rather than six provides an even greater ability to distinguish one conformation from another. Lastly, we will expand the bonds of inter­est to be the core C—N, core C—O, N—C_tol­yl_, and O—C_naphth­yl_ bonds, and assign descriptors to each in that order. Thus, the tolnaftate mol­ecule shown in Fig. 1 ▸ is the (+*ap*,−*sp*,+*ac*,−*ac*) conformer, while its enanti­omer would be uniquely described as the (−*ap*,+*sp*,−*ac*,+*ac*) conformer.

### Inter­molecular inter­actions and packing   

**Figure 2:**
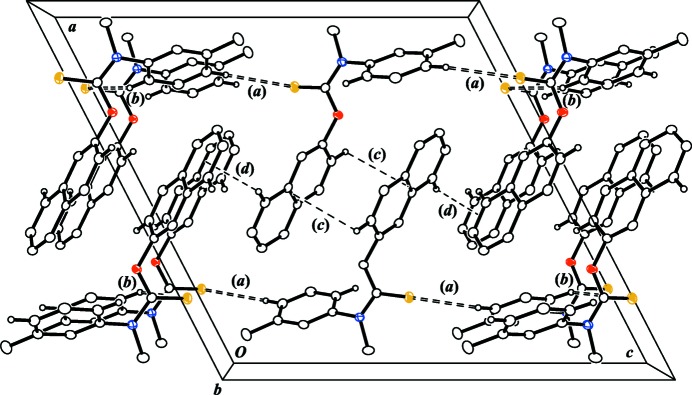
A unit-cell plot for (I) viewed down the *b* axis and showing the inter­molecular inter­actions present. (*a*) C16—H16⋯S11^i^, (*b*) C18—H⋯S11^ii^, (*c*) C3—H3⋯*X*1 and (*d*) C8—H8⋯*X*2. Displacement ellipsoids are drawn at the 50% probability level and only H3, H8, H16 and H18 and their equivalents are shown for clarity. *X*1 and *X*2 correspond to the points of closest contact between H3 and H8 to neighboring aromatic π planes, respectively. [Symmetry codes: (i) *x*, −*y* + 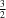, *z* + 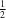; (ii) *x*, *y* + 1, *z*.]

A unit cell and packing diagram for (I) is shown in Fig. 2 ▸. The distances and angles for the four crystallographically unique inter­molecular inter­actions are given in Table 2 ▸. Two of the inter­actions are traditional resonance-induced C*sp* ^2^—H⋯S=C hydrogen bonds (Allen *et al.*, 1997 ▸), *i.e.* C16—H16⋯S11^i^ and C18—H18⋯S11^ii^ [symmetry codes: (i) *x*, −*y* + 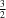, *z* + 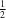; (ii) *x*, *y* + 1, *z*]. The observed H16⋯S11^i^ and H18⋯S11^ii^ distances are 2.98 and 2.93 Å, respectively, and are comparable to distances of 2.86–3.09 Å reported by others for C*sp* ^2^—H⋯S=C hydrogen bonds (Liu *et al.*, 2008 ▸; Omondi *et al.*, 2009 ▸). For C—H distances normalized to 1.089 Å, the H16⋯S11^i^ and H18⋯S11^ii^ distances are 2.86 and 2.81 Å, respectively, while the sum of the van der Waals radii for H and S is 3.00 Å (Bondi, 1964 ▸).

The other two inter­molecular inter­actions correspond to naphthyl-to-naphthyl C*sp* ^2^—H⋯π hydrogen bonds, *i.e.* C3—H3⋯*X*1 and C8—H8⋯*X*2. The first is an offset face-to-face hydrogen bond typically observed in π–π stacking, while the second is an edge-to-face hydrogen bond. The H3⋯*X*1 distance is 3.43 Å and is comparable to the value of 3.5 Å expected for a face-to-face hydrogen bond, while H8⋯*X*2 and C8—H8⋯*X*2 are 2.62 Å and 142°, and are in agreement with the values of 2.73 (13) Å and 148 (11)° expected for edge-to-face H⋯π and C*sp* ^2^—H⋯π, respectively (Takahashi *et al.*, 2001 ▸, 2010 ▸).

As shown in Fig. 2 ▸, the C*sp* ^2^—H⋯S=C inter­actions form two-dimensional (2D) networks of hydrogen bonds that are parallel to the (*h*00) family of planes at *x* = 0.2 and *x* = 0.8. Similarly, the C*sp* ^2^—H⋯π inter­actions form a separate 2D network of hydrogen bonds also parallel to the (*h*00) family of planes but at *x* = 0.5. The end result is a packing structure reminiscent of an inter­digitated *lipid bilayer* with the heteroatoms and polar bonds positioned near the outer surfaces and the nonpolar naphthyl substituents sandwiched in the inter­ior of the bilayer.

### Web theoretical conformations   

While the CSD mentioned above is *the world’s repository for small-mol­ecule crystal structures* with over 900,000 curated entries, its database of experimental 3D coordinates is miniscule compared to databases providing *theoretical* 3D coordinates. For example, the PubChem database currently contains 96,396,575 compounds and 3D coordinates for over 88.5 *million* mol­ecules (Kim *et al.*, 2016 ▸)! Freely available online theoretical 3D coordinates are largely a 21st Century global phenomenon, *e.g.* PubChem was launched in 2004, Mol-Instincts in 2006, and ATB in 2011, and are located in the USA, South Korea, and Australia, respectively. We will also mention 3DChem in the UK. With a holding of just 508 compounds, 3DChem is not as comprehensive as PubChem, Mol-Instincts or ATB, but it did list tolnaftate among its April 2017 *Mol­ecules of the Month* showcasing of anti­fungal agents.

**Figure 3:**
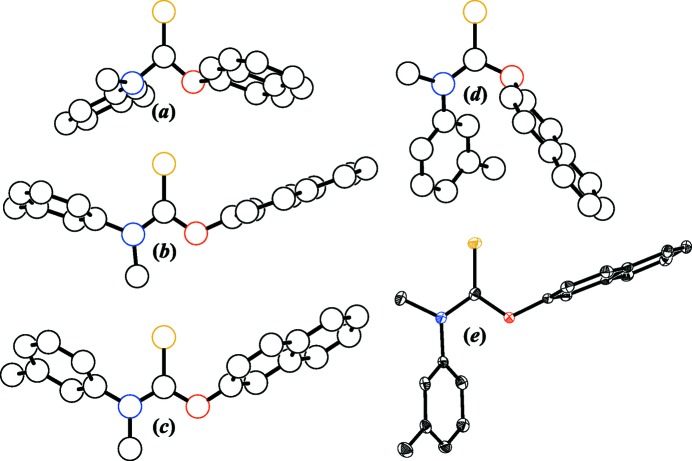
Online theoretical tolnaftate models *versus* the experimental X-ray structure. (*a*) 3DChem, (*b*) ATB, (*c*) Mol-Instincts, (*d*) PubChem, and (*e*) this work. Each mol­ecule is viewed normal to its central CNOS plane, H atoms have been removed for clarity, open circles are drawn to a common arbitrary size, and displacement ellipsoids are depicted at the 50% probability level. The downloaded ATB coordinates have been inverted to facilitate comparisons with the other models.

A visual comparison of online theoretical tolnaftate models *versus* our X-ray structure is shown in Fig. 3 ▸, and selected distances and angles are provided in Table 3 ▸. The conformations for the tolnaftate mol­ecules in Fig. 3 ▸ are (+*sc*,+*sc*,+*sp*,+*sc*), (+*sp*,+*sp*,+*ac*,−*ac*), (+*sp*,+*sp*,+*ac*,−*ac*), and (+*ac*,+*ac*,−*sp*,+*ap*) for the 3DChem, ATB, Mol-Instincts, and PubChem models, respectively, while the experimentally observed conformation for (I) is (+*ap*,−*sp*,+*ac*,−*ac*). The ATB and Mol-Instincts models are visually similar, but are strikingly different from the 3DChem and PubChem models. None of the theoretical models are a match to our X-ray structure. This is not unexpected since most theoretical models ignore inter­molecular inter­actions and packing forces such as those described above in §3.2. That having been said, the mismatch among the theoretical models themselves is probably of greater concern than their mismatch to (I).

The distances and angles in Table 3 ▸ reveal the discrepancies in the online theoretical structures. The 3DChem C—N bond at 1.468 Å is a significant outlier. The PubChem C—O bond at 1.432 Å is unusually long. The 3DChem C—S bond is uncomfortably short at 1.595 Å, while the opposite is true for the Mol-Instincts C—S bond at a lengthy 1.712 Å. The 3DChem N—C—O angle is alarmingly acute at 99.0° and its O—C—S angle is alarmingly obtuse at 131.1°. The Mol-Instincts N—C—O, N—C—S, and O—C—S angles are all 120.0°, a highly improbable occurrence, suggesting that that model was likely minimized with constraints. The PubChem O—C—S angle is also an outlier at a meager 115.3°. These nonsensical distances and angles for just the CNOS cores alone suggest that the 3DChem, Mol-Instincts and PubChem models are somewhat suspect. This is not to say that these models are invalid, as they may represent local minima on the tolnaftate potential energy landscape, but the unreasonableness of their CNOS core geometries suggests that attempting to rank them on a common relative energy scale is not worth the effort. Rather, we will simply say that the 3D coordinates from ATB (Malde *et al.*, 2011 ▸) appear to be the most robust set among this small sampling of theoretical tolnaftate models.

Taken as a whole, all of these observations suggest that the current standards and guidelines for online theoretical 3D models and coordinates are rather loose, and that the validation methods used by website providers for assessing the structural reasonableness of their optimized mol­ecules are less than fully adequate. Individuals in our targeted audience of nonscience professionals should therefore consider *any* 3D model or coordinates that they download from the web to be potentially suspect unless clearly demonstrated otherwise, *i.e.* we encourage them to critically examine the geometrical attributes (distances, angles *etc.*) of those models or seek help from others to do so, if need be.

### Peer-reviewed theoretical conformations   

**Figure 4:**
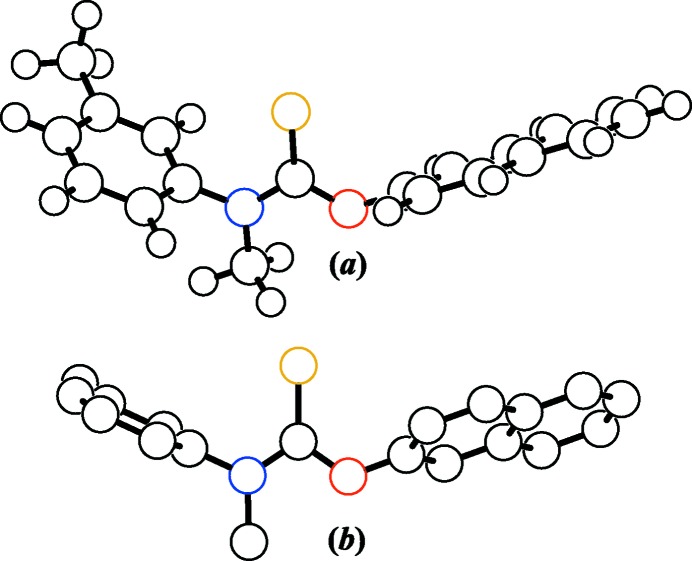
A peer-reviewed theoretical tolnaftate model. (*a*) The Dhas model (simulated) oriented approximately as published (Dhas *et al.*, 2011 ▸) and (*b*) viewed normal to the central CNOS plane. Open circles are of arbitrary size and H atoms have been removed from (*b*) for clarity.

For completeness, we are aware of two additional theoretical tolnaftate models. The first of these was published by Joe and co-workers as part of a vibrational analysis of the tolnaftate IR and Raman spectra (Dhas *et al.*, 2011 ▸). A *simulation* of their model is shown in Fig. 4 ▸. The conformation depicted in Fig. 4 ▸ was generated with the freeware mol­ecular editor *Avogadro 1.2.0n*, downloaded from https://avogadro.cc/ (Hanwell *et al.*, 2012 ▸), and adjusted until there was a reasonable match to Fig. 1 ▸ in the Dhas 2011 ▸ publication. The Dhas model is clearly a variant of the ATB and Mol-Instincts theoretical models, *i.e.* (+*sp*,+*sp*,+*ac*,−*ac*), and not a match to our experimentally observed (+*ap*,−*sp*,+*ac*,−*ac*) conformation. Our X-ray results are particularly relevant to this 2011 paper since their IR spectrum was taken on a solid-state tolnaftate sample in KBr. Since our study unequivocally establishes that the solid-state conformation of tolnaftate is (+*ap*,−*sp*,+*ac*,−*ac*) and not (+*sp*,+*sp*,+*ac*,−*ac*), their vibrational analysis based on the latter conformation is unlikely to be valid. For their published results to be valid, their sample would have to be a tolnaftate polymorph with the mol­ecules in the (+*sp*,+*sp*,+*ac*,−*ac*) conformation, which is highly improbable. To our knowledge, there is no powder diffraction or DSC evidence that tolnaftate polymorphs exist. Significant variations in powder pattern peak intensities have been observed, but such observations are completely attributable to preferred orientation effects without the need to invoke polymorphism. Hence, the calculated wavenumbers and *all* other computed qu­anti­ties based on their use of a (+*sp*,+*sp*,+*ac*,−*ac*) model should be considered suspect.

**Figure 5:**
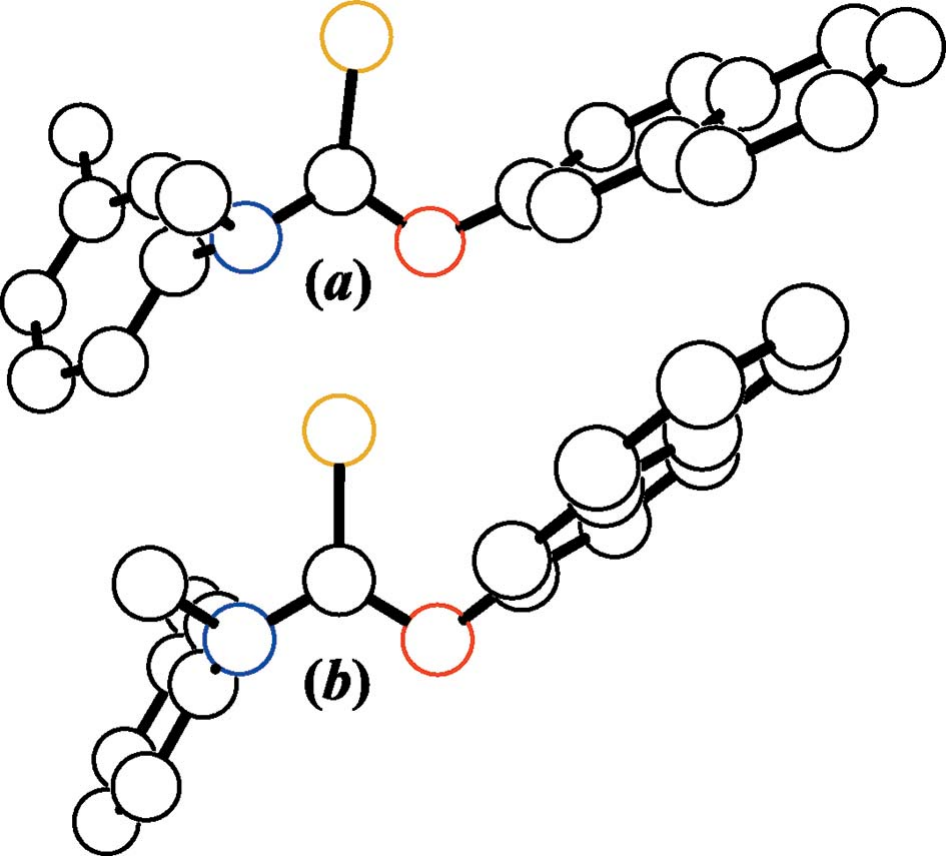
A second peer-reviewed theoretical tolnaftate model. (*a*) The Sun model (simulated) oriented approximately as published (Sun *et al.*, 2017 ▸) and (*b*) viewed normal to the central CNOS plane. Open circles are of arbitrary size and H atoms have been removed for clarity.

The second peer-reviewed theoretical tolnaftate model that we are aware of is that published by Sun and Liu and co-workers as part of a study on the *in silico* docking of tolnaftate into the active site of squalene epoxidase (Sun *et al.*, 2017 ▸). A *simulation* of their model is shown in Fig. 5 ▸. As with the 2011 paper above, no 3D coordinates were provided, so we used *Avogadro 1.2.0n* to approximate the theoretical tolnaftate model in Fig. 4 ▸ of the Sun 2017 ▸ publication. The Sun model appears to be a variant of the 3DChem model and is also not a match to our experimentally observed X-ray structure. Further, the CNOS core depicted in their Fig. 4 ▸ exhibits significant abnormalities, *e.g.* their tolnaftate C—S and C—N bonds seem unrealistically long, the C—O bond too short and the N—C—S angle overly obtuse. Their tolnaftate N atom having a pyramidal geometry is also highly unprecedented. Moreover, their Fig. 4 ▸ also indicates that the binding of liranaftate, a related and sterically bulkier thio­carbamate, to squalene epoxidase occurs without comparable distortion to its CNOS core. Unfortunately, even their liranaftate model is not without peculiarities, *e.g.* the pyramidalization of the aromatic C4a and C8a atoms in the *O*-5,6,7,8-tetra­hydro­naphthalen-2-yl moiety is chemically and structurally unrealistic. All of these observations suggest that both of their theoretical models, *i.e.* for tolnaftate and for liranaftate, are probably indefensible and that a follow-up study may be needed to ascertain whether the findings and conclusions of their 2017 study are valid or not.

What this comparison of our experimental model to peer-reviewed theoretical models reveals is that the standards and guidelines for publishing theoretical structures are currently less rigorous than those for publishing crystal structures. *Acta Crystallographica*, where Density Functional Theory (DFT) and various other theoretical results are increasingly being showcased, should consider implementing policies that will safeguard against questionable theoretical models reaching print. Insisting that 3D coordinate files *must be* submitted as supplementary material for theoretical models (and not just for X-ray structures) would greatly facilitate the peer-review process by referees and editors, and any subsequent scientific inquiries by readers.

## Summary and closing comments   

The take-home message for amateur scientists and science enthusiasts is that opportunities for scientific adventures and discoveries in a home setting do indeed exist and may even be publishable. All of the wet chemistry presented in this paper were done in a common household kitchen and all of the structure solution, refinement, and manuscript preparation were carried out with readily available freeware using public computers in local libraries. Exploring science in the 21st Century can be that simple. Also, be willing to investigate opportunities at local colleges and universities where specialized equipment like an X-ray diffractometer might be available through various outreach programs. Adult supervision and guidance are, of course, encouraged for any projects involving minors or other pre-college individuals.

Our message for academic and industrial colleagues is that the long overdue X-ray structure of tolnaftate is now available, and that crystals of tolnaftate contain a 50:50 mixture of (+*ap*,−*sp*,+*ac*,−*ac*) and (−*ap*,+*sp*,−*ac*,+*ac*) conformers. The broad implication and significance of this *experimental* finding is that it calls into question, either directly or indirectly, the results and conclusions from prior *theoretical* models for tolnaftate. Notably, the vibrational analysis, natural bonding orbital (NBO) analysis, and predicted electronic absorption spectrum and associated computed qu­anti­ties based on an alternate (+*sp*,+*sp*,+*ac*,−*ac*) conformation needs to be revisited and their actual numeric contributions to the bioactivity of tolnaftate reassessed. Similarly, our crystal structure suggests that a recent study on the binding mode of tolnaftate to squalene epoxidase by *in silico* methods is also in need of being revisited. It seems likely that the unusual tolnaftate (and liranaftate) conformation in that study was introduced at the ligand preparation stage. However, we cannot rule out with certainty whether the irregularities occurred at the docking stage, instead, or are due to other problems with the modeling methodology that was employed. Regardless, our kitchen-sink science argues that we do not yet know all that there is to know about the spectroscopy and enzyme-ligand inter­actions of this deceptively simple and historic anti­fungal compound.

## Supplementary Material

Crystal structure: contains datablock(s) global, I. DOI: 10.1107/S2053229618013591/yp3169sup1.cif

Structure factors: contains datablock(s) I. DOI: 10.1107/S2053229618013591/yp3169Isup2.hkl

Supporting information file. DOI: 10.1107/S2053229618013591/yp3169Isup3.cdx

Supporting information file. DOI: 10.1107/S2053229618013591/yp3169Isup4.cml

CCDC reference: 1869447

## Figures and Tables

**Table 1:** Experimental details

Crystal data	
Chemical formula	C_19_H_17_NOS
*M*_r_	307.39
Crystal system, space group	Monoclinic, *P*2_1_/*c*
Temperature (K)	100
*a*, *b*, *c* (Å)	17.0498 (11), 5.7778 (4), 18.1012 (11)
β (°)	117.3590 (12)
*V* (Å^3^)	1583.70 (18)
*Z*	4
Radiation type	Mo *K*α
μ (mm^−1^)	0.21
Crystal size (mm)	0.29 × 0.18 × 0.07

Data collection	
Diffractometer	Bruker Kappa APEXII DUO
Absorption correction	Numerical (*SADABS*; Bruker, 2014 ▸)
*T*_min_, *T*_max_	0.906, 1.000
No. of measured, independent and observed [*I* > 2σ(*I*)] reflections	6795, 3706, 2935
*R*_int_	0.020
(sin θ/λ)_max_ (Å^−1^)	0.659

Refinement	
*R*[*F* ^2^ > 2σ(*F* ^2^)], *wR*(*F* ^2^), *S*	0.038, 0.093, 1.04
No. of reflections	3706
No. of parameters	202
H-atom treatment	H-atom parameters constrained
Δρ_max_, Δρ_min_ (e Å^−3^)	0.29, −0.27

Computer programs: *APEX2* (Bruker, 2014 ▸), *SAINT* (Bruker, 2013 ▸), *SADABS* (Bruker, 2014 ▸), *XPREP* (Bruker, 2014 ▸), *SHELXT2018* (Sheldrick, 2015*a* ▸), *SHELXL2018* (Sheldrick, 2015*b* ▸), *SHELXTL* (Sheldrick, 2008 ▸) and *publCIF* (Westrip, 2010 ▸).

**Table 2:** Hydrogen-bond geometry (Å, °) for (I) *X*1 and *X*2 are points on neighboring naphthalene π planes from which normals are drawn to H3 and H8, respectively (Takahashi *et al.*, 2001 ▸).

*D*—H⋯*A*	*D*—H	H⋯*A*	*D*⋯*A*	*D*—H⋯*A*
C3—H3⋯*X*1	0.95	3.43	3.56	90
C8—H8⋯*X*2	0.95	2.62	3.42	142
C16—H16⋯S11^i^	0.95	2.98	3.8637 (16)	154
C18—H18⋯S11^ii^	0.95	2.93	3.7744 (16)	149

Symmetry codes: (i) *x*, −*y* + 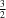, *z* + 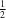; (ii) *x*, *y* + 1, *z*.

**Table 3:** Selected distances and angles (Å, °) for a sampling of online theoretical tolnaftate models *versus* the experimental X-ray structure (I) Websites: http://3dchem.com/, https://atb.uq.edu.au/, https://www.molinstincts.com/home/index/, and https://pubchem.ncbi.nlm.nih.gov/.

Parameter	3DChem	ATB	Mol-Instincts	PubChem	This work
C—N	1.468	1.346	1.348	1.405	1.3444 (19)
C—O	1.384	1.359	1.348	1.432	1.3556 (18)
C—S	1.595	1.683	1.712	1.677	1.6567 (15)
N—C—O	99.0	109.5	120.0	117.2	110.39 (13)
N—C—S	129.9	126.3	120.0	127.5	125.11 (12)
O—C—S	131.1	124.1	120.0	115.3	124.48 (11)
S—C—N—C_tol­yl_	84.4	0.9	4.6	148.4	179.98 (12)
S—C—O—C_naphth­yl_	56.7	6.5	6.0	121.4	−5.5 (2)
C—N—C—C	19.9	103.8	113.6	−30.0	121.42 (16)
C—O—C—C	33.6	−97.0	−112.2	150.1	−95.70 (17)
